# Loci Associated With Antibody Response in Feral Swine (*Sus scrofa*) Infected With *Brucella suis*

**DOI:** 10.3389/fvets.2020.554674

**Published:** 2020-11-25

**Authors:** Courtney F. Pierce, Vienna R. Brown, Steven C. Olsen, Paola Boggiatto, Kerri Pedersen, Ryan S. Miller, Scott E. Speidel, Timothy J. Smyser

**Affiliations:** ^1^United States Department of Agriculture, Animal and Plant Health Inspection Service, Wildlife Services, National Wildlife Research Center, Fort Collins, CO, United States; ^2^Department of Animal Sciences, Colorado State University, Fort Collins, CO, United States; ^3^United States Department of Agriculture, Animal and Plant Health Inspection Service, Wildlife Services, National Feral Swine Damage Management Program, Fort Collins, CO, United States; ^4^United States Department of Agriculture, Agricultural Research Service, Infectious Bacterial Diseases, National Animal Disease Center, Ames, IA, United States; ^5^United States Department of Agriculture, Animal and Plant Health Inspection Service, Wildlife Services, Raleigh, NC, United States; ^6^United States Department of Agriculture, Animal and Plant Health Inspection Service, Veterinary Services, Center for Epidemiology and Animal Health, Fort Collins, CO, United States

**Keywords:** *Brucella suis*, brucellosis, disease spillover, feral swine, GWAS, SNP, *Sus scrofa*

## Abstract

Feral swine (*Sus scrofa*) are a destructive invasive species widespread throughout the United States that disrupt ecosystems, damage crops, and carry pathogens of concern for the health of domestic stock and humans including *Brucella suis*—the causative organism for swine brucellosis. In domestic swine, brucellosis results in reproductive failure due to abortions and infertility. Contact with infected feral swine poses spillover risks to domestic pigs as well as humans, companion animals, wildlife, and other livestock. Genetic factors influence the outcome of infectious diseases; therefore, genome wide association studies (GWAS) of differential immune responses among feral swine can provide an understanding of disease dynamics and inform management to prevent the spillover of brucellosis from feral swine to domestic pigs. We sought to identify loci associated with differential antibody responses among feral swine naturally infected with *B. suis* using a case-control GWAS. Tissue, serum, and genotype data (68,516 bi-allelic single nucleotide polymorphisms) collected from 47 feral swine were analyzed in this study. The 47 feral swine were culture positive for *Brucella* spp. Of these 47, 16 were antibody positive (cases) whereas 31 were antibody negative (controls). Single-locus GWAS were performed using efficient mixed-model association eXpedited (EMMAX) methodology with three genetic models: additive, dominant, and recessive. Eight loci associated with seroconversion were identified on chromosome 4, 8, 9, 10, 12, and 18. Subsequent bioinformatic analyses revealed nine putative candidate genes related to immune function, most notably phagocytosis and induction of an inflammatory response. Identified loci and putative candidate genes may play an important role in host immune responses to *B. suis* infection, characterized by a detectable bacterial presence yet a differential antibody response. Given that antibody tests are used to evaluate brucellosis infection in domestic pigs and for disease surveillance in invasive feral swine, additional studies are needed to fully understand the genetic component of the response to *B. suis* infection and to more effectively translate estimates of *Brucella* spp. antibody prevalence among feral swine to disease control management action.

## Introduction

In the United States (U.S.) there are ~6 million invasive feral swine (*Sus scrofa*), which are defined as any released or escaped domestic pigs, Eurasian wild boars, or hybrids of the two (1, 2). Both the abundance of feral swine and extent of the geographic range have increased rapidly over the past 30 years due to the high reproductive potential of the species, limited predation pressure, abundance of food (both native flora and fauna and agricultural products), and human-mediated introduction into uninvaded habitats (3, 4). As range and abundance have increased, so too have the economic and ecological costs of feral swine. Feral swine negatively impact the aesthetic and cultural value of landscapes, with costs to tourism and silvicultural sectors; however, direct costs to agriculture are most notable. In a survey of 10 of the 38 states with established populations, feral swine cause an estimated $190 million in crop damages annually (5). Feral swine also damage pastures through rooting and trampling behaviors that kill desired plant species and allow unpalatable species to quickly spread (6). Among the broader costs associated with the expansion of this invasive species, feral swine serve as an important reservoir for a number of pathogens (e.g., *Brucella* spp., pseudorabies virus, and trichinella) with the potential for spillover to livestock, humans, companion animals, and wildlife (7–9).

Of the pathogens commonly detected in feral swine, swine brucellosis is among the most important, in large part because of its broad host specificity (10). *Brucella* spp. are facultative intracellular bacteria that primarily infect phagocytic cells following infection of the host. Typical pathogen recognition involves actin cytoskeletal remodeling in which membrane protrusions extend and uptake the stimulatory particle, thus generating a phagosome (11). The phagosome typically matures and fuses with the lysosome to create a phagolysosome, which destroys microorganisms. However, *Brucella* spp. have been shown to disrupt this process within the host cell by modifying the original phagosome into a membrane bound vesicle, referred to as the *Brucella*-containing vacuole [BCV; (12)], which prevents lysosome fusion. The BCV moves along the endocytic pathway developing membrane markers associated with both the late endosome and the endoplasmic reticulum. The Type IV secretion system (T4SS) Vir B has been demonstrated to use effector proteins to modulate secretory trafficking and promote bacterial pathogenesis (13, 14). Knowledge of the infection pathway and kinetics of *Brucella* spp. is crucial for interpreting diagnostic results and understanding host pathophysiology.

Several species of *Brucella* have been isolated from feral swine, including *suis, abortus*, and *microti* (15–17), respectively. However, *Brucella suis* is the only species to cause systemic or generalized infection in swine and can lead to reproductive failure (18). Through federal, state, tribal, and industry partnerships, brucellosis was eradicated from domestic pigs in the U.S. in 2011 (19–21). However, brucellosis remains prevalent among feral swine, as disease surveillance efforts throughout the invaded range within the U.S. documented an apparent *Brucella* spp. antibody prevalence of 4.3% (8). Prevalence rates vary geographically, with highest antibody prevalence observed among Hawaii, South Carolina, and Alabama [14.4, 11.6, and 10.8%, respectively; (21)]. The high prevalence of *B. suis* in feral swine poses a risk of reemergence of this bacterial pathogen in domestic pigs. Approximately 36.5% of commercial swine facilities, maintaining 11.3% of the nation's pork inventory, are located in regions where feral swine are present (22). Further, monitoring of feral swine fitted with Global Positioning System (GPS) telemetry collars has demonstrated direct interactions with livestock, with pasture-raised pigs at greatest risk for *B. suis* exposure (23). United States pork production and processing is estimated to contribute $39 billion to the gross domestic product (24); however, reemergence of *B. suis* among commercial swine could result in tremendous economic losses and trade restrictions for pork producers (25).

Although swine are the primary host for *B. suis*, this bacterium can infect a number of other species, including cattle (26). *Brucella suis* generally causes few clinical signs and is not believed to be transmitted among cattle (27). However, infection of lactating cows results in shedding of bacteria in milk and the development of antibodies, which cannot be differentiated from *B. abortus* antibodies with current diagnostic serological tests (28). Thus, bacterial spillover from feral swine to cattle creates significant diagnostic challenges in addition to posing a public health risk via consumption of raw milk (27). Furthermore, as a zoonotic pathogen, *B. suis* has significant public health implications with human cases of brucellosis in the U.S. most often associated with exposure through feral swine hunting and field dressing (29).

Immunity against intracellular pathogens, including *Brucella* spp. relies on the induction of cell-mediated immunity, primarily interferon gamma (IFN-γ)-producing CD4^+^ T cells, or T helper 1 (T_H_1) responses. These responses can be measured from blood samples; however, they require cellular isolation, *in vitro* antigen stimulation, and can take days to obtain results. Therefore, cell-mediated responses are not frequently used for diagnostic purposes. Rather for *Brucella suis*, as with other *Brucella* spp., infection is diagnosed using antigen and antibody assays. However, culture and serological diagnostics for *B. suis* often produce conflicting results (9, 28, 30, 31). *Brucella* lipopolysaccharides (LPS) are generally less toxic than those of other gram-negative bacteria, which are potent stimulators of innate immune responses through pattern-recognition receptors (32). However, the O-polysaccharide of the *Brucella* LPS is immunodominant for humoral responses during infection in most natural hosts. Therefore, the O-polysaccharide is the primary antigen used in most serologic tests for detecting infection with *B. abortus, B. melitensis*, or *B. suis* (33). In infected swine, brucellosis serologic tests have lower sensitivity than in other host species such that serologic testing is usually evaluated on a herd, rather than an individual basis (26). Low sensitivity of the assay may be due to the fact that most serologic tests were developed and validated for cattle infected with *B. abortus* and there may be structural differences between *B. abortus* and *B. suis* LPS (34).

Genetic variation has been documented to play a role in immune kinetics for numerous species and diseases (35–39). For example, genetic variation in host resistance or tolerance has been reported (scientifically and anecdotally) in swine for African swine fever, foot-and-mouth disease, atrophic rhinitis, pseudorabies, and brucellosis (36, 39). Similarly, we sought to evaluate whether genetic factors may define variation in seroconversion to *B. suis* infection with implications for the reliability of antibody tests used for monitoring disease prevalence among feral swine (9). We conducted case-control genome-wide association studies (GWAS) in which allele frequencies of single nucleotide polymorphisms (SNP) spanning the genome were evaluated to identify loci associated with *B. suis* seroconversion in wild-caught, naturally infected feral swine.

## Materials and Methods

### Sample Collection

Tissue (submandibular, parotid, medial retropharyngeal, tracheobronchial, gastrohepatic, axillary or inguinal lymph nodes, spleen, and reproductive tract) and serum samples were collected from 376 feral swine at two Texas abattoirs in 2015 as described by Pedersen et al. (9). Tissue cultures were conducted at the U.S. Department of Agriculture's Agricultural Research Service in Ames, Iowa and at the College of Veterinary Medicine at Texas A&M University in College Station, Texas. If any of the tissue samples contained *Brucella* spp., the animal was considered culture positive. In accordance with established protocols, the following eight independent serological assays were completed for each animal at the National Veterinary Services Laboratories (NVSL): (1) buffered antigen plate agglutination test (BAPA), (2) competitive enzyme-linked immunosorbent assay (cELISA), (3) complement fixation, (4) fluorescence polarization assay (FPA), (5) the rivanol test, (6) plate agglutination, (7) tube agglutination, and (8) card test. Given the limitations of these serological assays, feral swine were considered seropositive for *B. suis* if two or more of these assays were positive (9). Forty-nine feral swine were culture positive for *Brucella* spp. and, of these, 16 were considered antibody positive (Table 1). Similarly, individuals with one or zero positive results from the 8 serological assays were classified as negative. In this study, animals that were both culture positive and seropositive were defined as cases whereas animals that were culture positive but seronegative were defined as controls.

**Table 1:** Feral swine samples collected at two Texas abattoirs that were culture positive for *B. suis*.

		**Sex**	
**Serological Result**	**Age**	**Male**	**Female**
Positive	Adult	10	5
	Sub-adult	1	0
Negative	Adult	14	11
	Sub-adult	3	5

Misclassification of the serological status of an animal could result from two potential sources of error—error due to diagnostic test performance or the delay in immune response once infected resulting in a false negative result. The probability of an individual being misclassified as negative when positive due to diagnostic test performance was calculated as the combined probability of seven or eight false negative serological results using the lowest previously published sensitivity value for each serological diagnostic test (40, 41). False negative error resulting from the delay in immune response was assessed using data describing the serum antibody response of an infected animal to the eight diagnostic tests [complete methods available in Supplementary Material; (42, 43)]. Bayesian generalized additive models were fit to these serological response data for each of the eight diagnostic tests resulting in the predicted increase and variation in serological response by day after infection. Using these posterior predictions for serological response, two simulations were conducted to determine the probability that an individual animal will test positive. First, the probability of at least two positive test results for each day post-infection were simulated. Second, because the time of infection is unknown for sampled animals in this study the probability that our sample included serological true positive and false negative animals was conservatively calculated assuming infection occurred within 120 days of being sampled.

### Genotype Data

Bi-allelic SNP genotypes for feral swine were generated using the GeneSeek Genomic Profiler for Porcine bead array [68,516 loci; Illumina BeadChip microarrays (San Diego, California) licensed exclusive to GeneSeek, Neogen Corporation (Lincoln, Nebraska); (44)]. Single nucleotide polymorphisms were mapped to the Sscrofa11.1 reference genome assembly (45) and non-autosomal loci were removed, leaving 62,128 loci available for analysis. We then used SNP & Variation Suite (SVS; Golden Helix, Bozeman, Montana) and PLINK 1.9 (46) to implement standard quality control measures for GWAS analysis of SNP genotypes, specifically pruning loci with call rates ≤0.90, minor allele frequency <0.01, Hardy-Weinberg Equilibrium <1 × 10^−6^, and removing samples with call rates ≤0.90 and heterozygosity rate ±3 standard deviations from the mean. After quality control, 47 swine (16 cases [seropositive]/31 [seronegative] controls) and 53,162 SNP were retained for analysis. As an additional quality control step, we screened these 47 individuals for close familial relationships (identical by descent [pi-hat] estimate >0.375). Finding no dyads that exceeded this threshold, all 47 individuals were retained.

### Statistical Analysis

A single-locus mixed model GWAS was evaluated using the efficient mixed-model association eXpedited (EMMAX) methodology in SVS (Golden Helix, Bozeman, Montana). Association analyses were conducted using three genetic models: additive, dominant, and recessive. Given a bi-allelic locus with two alleles (*A* and *a*), the additive model assumes that there is a linear increase in disease risk with each copy of the *A* allele; thus, the increase in disease risk from *aa* to *Aa* would again be doubled among *AA* homozygotes. The recessive model assumes that two copies of the *A* allele increases the risk of disease whereas the dominant model assumes that one or more *A* alleles increases the risk of disease (47, 48). The mixed model was as follows:

$$\begin{array}{l} y=X\beta +Z\mu +\varepsilon \end{array}$$

where, *y* was a vector of observations, *X* was a matrix of fixed effects, β was a vector of fixed effects to be estimated, Z was a matrix relating random effects to the observations in *y*, μ was a vector of random effects to be estimated, and ε was a vector of residual errors. It was assumed that $\sigma_{\mu }^{2}$ = $\sigma_{a}^{2}K$ and $\sigma_{\varepsilon }^{2}$ = $\sigma_{e}^{2}I$; therefore, $\sigma_{y}^{2}$ = $\sigma_{g}^{2}ZKZ\prime +\;\sigma_{e}^{2}I$ (49). K was a pre-computed genomic relationship matrix to account for population structure. Age (sub-adult vs. adult), sex, facility, and month in which the samples were collected were examined as potential fixed effects using an Akaike Information Criterion (AIC) approach (50). After screening fixed effects with AIC, only age was retained for additional model development. Association test statistics can be inflated by underlying population stratification (i.e., genetic structure associated with systematic differences in allele frequencies among genetic (sub)populations included in the sample) (51); therefore, genomic inflation factors (λ) were estimated to ensure that each model sufficiently corrected for population stratification. The genomic inflation factor is expressed as the median of the observed distribution of the test statistic divided by median of the expected distribution of the test statistic, where λ < 1.01 suggests small test statistic inflation, λ < 1.05 suggests moderate test statistic inflation, and λ > 1.1 suggests highly inflated test statistics (52). Our genomic inflation factors were 1.03, 1.00, and 1.04 for the additive, dominant, and recessive models, respectively; therefore, the models were deemed appropriate for subsequent analyses.

Single nucleotide polymorphisms were considered associated with *B. suis* seroconversion when their unadjusted *P* < 5 × 10^−5^, moderately associated when their unadjusted *P*-value fell between 5 × 10^−7^ and 1 × 10^−5^, and strongly associated when unadjusted *P* < 5 × 10^−7^ (53). To further examine the association between these loci and *B. suis* seroconversion, an odds ratio (OR) and 95% confidence interval were calculated for the major allele at each locus (54) as follows:

$$\begin{array}{l} OR=\frac{\left(AxD\right)}{\left(BxC\right)} \end{array}$$

where OR represents the odds ratio for the major allele at a given locus, A represents the number of major alleles within the cases, D represents the number of minor alleles within the control group, B represents the number of minor alleles within the cases, and C represents the number of major alleles in controls (55, 56).

### Functional Annotation

Reference sequence (RefSeq) gene transcripts, annotated by the National Center for Biotechnology Information (NCBI; *Sus scrofa* annotation release 106), were used to identify genes within 2 Mb (1 Mb upstream and 1 Mb downstream) of the candidate SNP (57–59). This interval was approximately twice the average haplotype block size (394.88 kb) for swine (60). The Pig Quantitative Trait Locus Database (Pig QTLdb; https://www.animalgenome.org/cgi-bin/QTLdb/SS/index) was queried to identify traits that were previously associated with the genes of interest. Kyoto Encyclopedia of Genes and Genomes (KEGG) Pathway Database (61) was used to identify disease-related pathways within *S. scrofa* that contained the candidate genes.

## Results

### Evaluation of the Misclassification of Serological Status

The combined probability of eight false negative serological results [*P*(x1,.,x8) = 2.03 × 10^−6^] or seven false negative results and one positive result [*P*(x1,.,x7) = 2.98 × 10^−4^] was low. The probability of accurately classifying an animal as serologically positive increased logistically with time since infection, approaching 1 at ~32 days post-infection. Assuming *B. suis* animals in this study were infected within 120 days of sampling, the probability that the sample included an animal misclassified as negative (false negative) was 0.13.

### Additive Model

**Figure 1:**
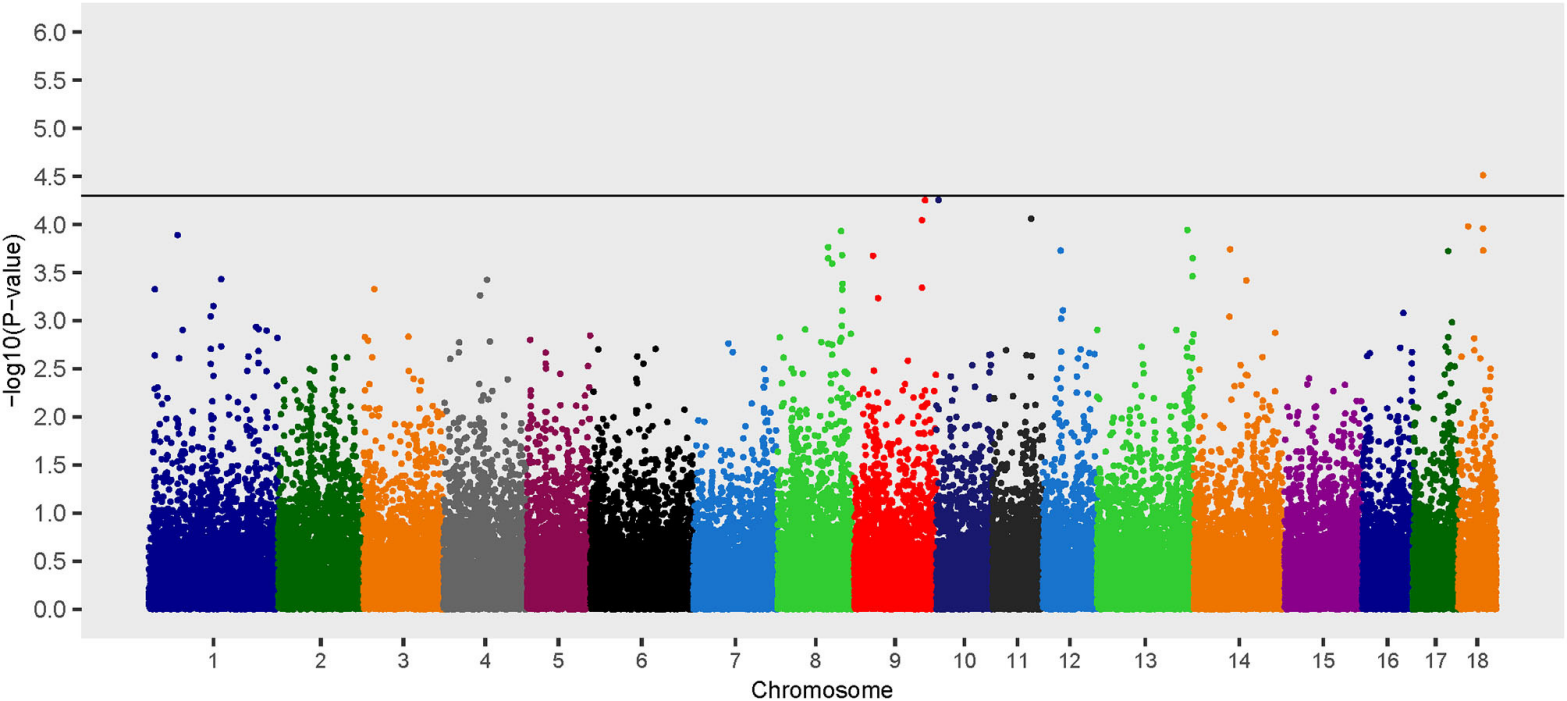
Manhattan plot of genome-wide association study for seroconversion following *Brucella suis* infection in feral swine using the EMMAX-GRM additive model. The black line [–log_10_(*P*-value) = 4.3] denotes an association with *B. suis*.

Three loci on chromosomes 9, 10, and 18 were associated with *B. suis* seroconversion (Figure 1). The odds ratio for the major allele at each locus was 0.10, 0.19, and 0.22 for rs339122633 (G), rs81477530 (C), and rs81469187 (A), respectively (Table 2). This suggests that the major alleles, in these three cases, were associated with a decreased likelihood of *B. suis* seroconversion. The 2 megabase (Mb) regions encompassing these three loci contained 26 annotated genes and 14 uncharacterized genes. A thorough review of candidate gene function, infection kinetics, and the immunologic response to *B. suis* infection reduced the number of putative candidate genes from 40 to three:(1) acyloxyacyl hydrolase (*AOAH*), (2) engulfment and cell motility 1 (*ELMO1*), and (3) prostaglandin synthase 2 (*PTGS2*). Locus rs339122633 was located within an intron of Hemicentin 1 (*HMCN1*); however, based on our current understanding of the functions of *HMCN1*, there is no obvious link to *B. suis* infection.

**Table 2:** Single nucleotide polymorphisms associated with seroconversion following *Brucella suis* infection in feral swine sampled at two Texas slaughterhouses.

**CHR^**a**^**	**RS ID^**b**^**	**Location^**c**^ (bp)**	**Model^**d**^**	***P*-value^**e**^**	**Odds Ratio^**f**^** **(95% C.I.)**	**Genes^**g**^**
4	rs80941838	76,298,218	Dominant	4.22 × 10^−5^	7.97 (2.50–25.42)	*ATP6V1H, CHCHD7, LOC100626876, LOC100627466, LOC102166699, LOC102166862, LOC106510084, LOC106510085, LOC110260293, LOC110260295, LOC110260483, LYN, LYPLA1, MOS, MRPL15, PENK, PLAG1, RGS20, RP1, RPS20, SDR16C5, SOX17, TCEA1, TGS1, TMEM68, **XKR4***
8	rs81404101	121,799,317	Dominant	3.59 × 10^−5^	0.23 (0.09–0.57)	*ADH1C, ADH4, ADH5, C8H4orf17, EIF4E, LOC100512795, LOC102160109, LOC102162205, LOC110261964, LOC110262184, LOC110262185, LOC110262186, LOC110262187, METAP1, MTTP, RAP1GDS1, STPG2, TRMT10A, **TSPAN5***
9	rs81413617	78,187,445	Recessive	2.29 × 10^−5^	0.30 (0.12–0.74)	*ASNS, C1GALT1, COL28A1, GLCCI1, ICA1, LOC102161092, LOC102161282, LOC106504961, LOC106504962, LOC110255476, **LOC110262233**, LOC110262296, MIOS, NXPH1, RPA3, TAC1, UMAD1*
9	rs339122633	126,945,973	Additive Dominant	5.60 × 10^−5^ 1.53 × 10^−6^	0.10 (0.03–0.35)	*C9H1orf27, EDEM3, FAM129A, **HMCN1**, IVNS1ABP, LOC100627489, LOC102159064, LOC106505035, LOC110262256, LOC110262257, PDC, PLA2G4A, PRG4, PTGS2, RNF2, SWT1, TPR, TRMT1L*
10	rs81477530	2,523,688	Additive	5.58 × 10^−5^	0.19 (0.07–0.48)	*BRINP3, LOC102166310, LOC102158399, LOC102166515, LOC110255734, LOC110255623, LOC110255624, RGS18*
12	rs81329776	17,391,711	Dominant	3.94 × 10^−5^	0.03 (0–0.22)	*ACBD4, ARF2, ARHGAP27, CDC27, CRHR1, DCAKD, FMNL1, GOSR2, HEXIM1, HEXIM2, ITGB3, KANSL1, LOC100516640, LOC100524336, LOC100624995, LOC100626147, LOC102159591, LOC102160528, LOC102165973, LOC102167583, LOC102167870, LOC106504254, LOC110255897, LOC110255991, LOC110255993, LOC110255994, LYZL6, MAP3K14, MAPT, MYL4, NMT1, NSF, PLCD3, PLEKHM1, RPRML, SPATA32, SPPL2C, TRNAA-CGC, WNT3, WNT9B*
18	rs81469187	38,333,198	Additive Dominant	3.08 × 10^−5^ 1.37 × 10^−5^	0.22 (0.09–0.0.54)	*ANLN, AOAH, DPY19L1, DPY19L2, EEPD1, ELMO1, HERPUD2, KIAA0895, LOC102161969, LOC110257555, LOC110257625, NPSR1, SEPT7, TBX20*
18	rs338961194	38,506,661	Dominant	3.69 × 10^−5^	0.25 (0.10–0.60)	*BMPER, LOC102163798, LOC106508214*

a *Autosome in which the locus was located according to Sscrofa 11.1 reference assembly*.

b *Reference SNP cluster identification assigned by the National Center for Biotechnology Information (NCBI)*.

c *Autosome position in which the locus was located according to Sscrofa 11.1 reference assembly*.

d *Genetic models examined: additive, dominant, and recessive*.

e *Unadjusted P-value for the SNP associated with B. suis*.

f *Odds ratio for the major allele of the locus associated with B. suis*.

g *Genes located within the 2 Mb region encompassing the SNP associated with B. suis. Bold font indicates that the SNP was located within the gene*.

#### AOAH

Expressed by monocytes, macrophages, neutrophils, and dendritic cells (62), the AOAH enzyme (encoded by the *AOAH* gene) removes secondary fatty acyl chains from lipopolysaccharides (LPS) on the outer membrane of Gram-negative bacteria, rendering the target bacterium immunologically inert (63, 64). In general, exposure to LPS induces a robust inflammatory response. This is followed by a period of tolerance that is believed to have evolved to minimize inflammation-induced damage during recovery from microbe exposure (64, 65). Once AOAH deacylates lipid A, the bioreactive center of the LPS, the ability to elicit an inflammatory response is greatly reduced (66, 67). Removal of fatty acid chains renders the LPS biologically inactive and reestablishes sensitivity for subsequent infections (64, 68).

As this SNP was associated with a decreased likelihood of seroconversion, modification of *AOAH* function may result in a phenotype in which the LPS of *B. suis* is not effectively processed in porcine phagocytes. Thus, LPS antigens, such as the O side-chain, may not be expressed as well with major histocompatibility complex (MHC) II molecules on the surface of antigen presenting cells, making them less available for immune recognition. As cell wall-derived polysaccharides and glycol lipids are not readily digested by lysososomal enzymes, they can be retained for long periods of time inside macrophages (69). Others have reported that *Brucella* LPS can form ternary complexes with MHC class II molecules that are sequestered in the macrodomains at the cell surface, which prevents immunologic presentation (70). The phenotype associated with the SNP may lead to more ternary complexes that decrease immunologic presentation by phagocytic cells, thereby facilitating reduced humoral responses.

#### ELMO1

The Engulfment and Cell Motility 1 gene encodes ELMO1, a host-signaling molecule in phagocytic and T cells that modulates cellular activities through activation of small Rac GTPases (71, 72). Rac cycles between active and inactive states based on binding of guanosine diphosphate (GDP) from guanine nucleotide exchange factors (GEF). The cytosolic region of brain angiogenesis inhibitor 1 (BAII, a receptor that recognizes the core carbohydrate of lipopolysaccharide) interacts with ELMO1 leading to Rac activation (72). This suggests a role for ELMO1 in innate immunity detection and response to bacterial pathogens (73). ELMO1 stimulated responses occur largely from within the cell after phagocytosis engulfment of the target, resulting in an amplified signal from the concentration of bacterial pathogen-associated molecular patterns within phagosomes (73).

In lymphocytes, ELMO1 appears to primarily function by regulating Dock2, a GEF for activating Rac GTPase (71). The Dock2.ELMO1 complex is essential for chemokine dependent migration of primary T and B cells and for key steps in the interactions between T cells and target cells. ELMO1 regulates polarization and migration in response to chemokine signals, but a lack of ELMO1 does not appear to impair normal homeostatic migration of peripheral T cells (71). After activation of the T cell receptor, a filamentous actin ring is part of the immunological synapse that forms between a T cell and the surface of a target cell. This provides a structural framework for effector function including directional secretion of cytokines and cytolytic factors (74). The Dock2.ELMO1 complex and Rac activation are important in formation of the filamentous actin ring as part of the T cell activation process. Dock2 has also been shown to be critical for T cell cytotoxicity (74), which may be important because of the intracellular localization of *Brucella* spp. Regulators of Rac GTPase signaling, such as ELMO1 and Dock2, continue to be of interest for understanding their roles in regulating T cell activation and function. As this SNP was also associated with a decreased likelihood of seroconversion, *ELMO1* may function to decrease the humoral response by reducing internalization of *Brucella* spp. into phagocytic cells, reducing degradation of bacterial antigens in phagosomes, and/or decreasing pro-inflammatory immune responses following infection.

#### PTGS2

The Prostaglandin-Endoperoxide Synthase 2 (*PTGS2*) gene encodes the PTGS2 enzyme that catalyzes the breakdown of arachidonic acid from cell wall phospholipids to bioactive eicosanoid compounds (75, 76). PTGS2 is expressed by a wide range of cells including macrophages, fibroblasts, vascular endothelial cells, and smooth muscle cells (77) and exposure of immune cells (dendritic or monocytes) to *B. abortus* or its LPS increases expression of prostaglandins (78, 79). Previous studies suggest that increased prostaglandin concentrations favor prolonged survival of *Brucella* within host cells. Cyclooxygenase inhibition reduces *Brucella* colonization in a murine model (78) and has been shown to have suppressive effects on several other immune-modulating effectors.

Prostaglandins are also potent immune modulators for T cells and can block T cell proliferation and promote cytokines associated with TH-2 responses (80, 81). As previous studies suggest that *Brucella* uses the prostaglandin pathway to subvert immune responses and enhance persistence in infected cells, it would be logical to hypothesize *PTGS2* affects immune responses through inhibiting the synthesis of prostaglandins, thereby reducing stimulation of TH-2 responses associated with antibody production. As animals with this SNP were culture positive, it would suggest that factors other than prostaglandin synthesis have critical roles in *in vivo* clearance of *B. suis* in pigs.

### Dominant Model

**Figure 2:**
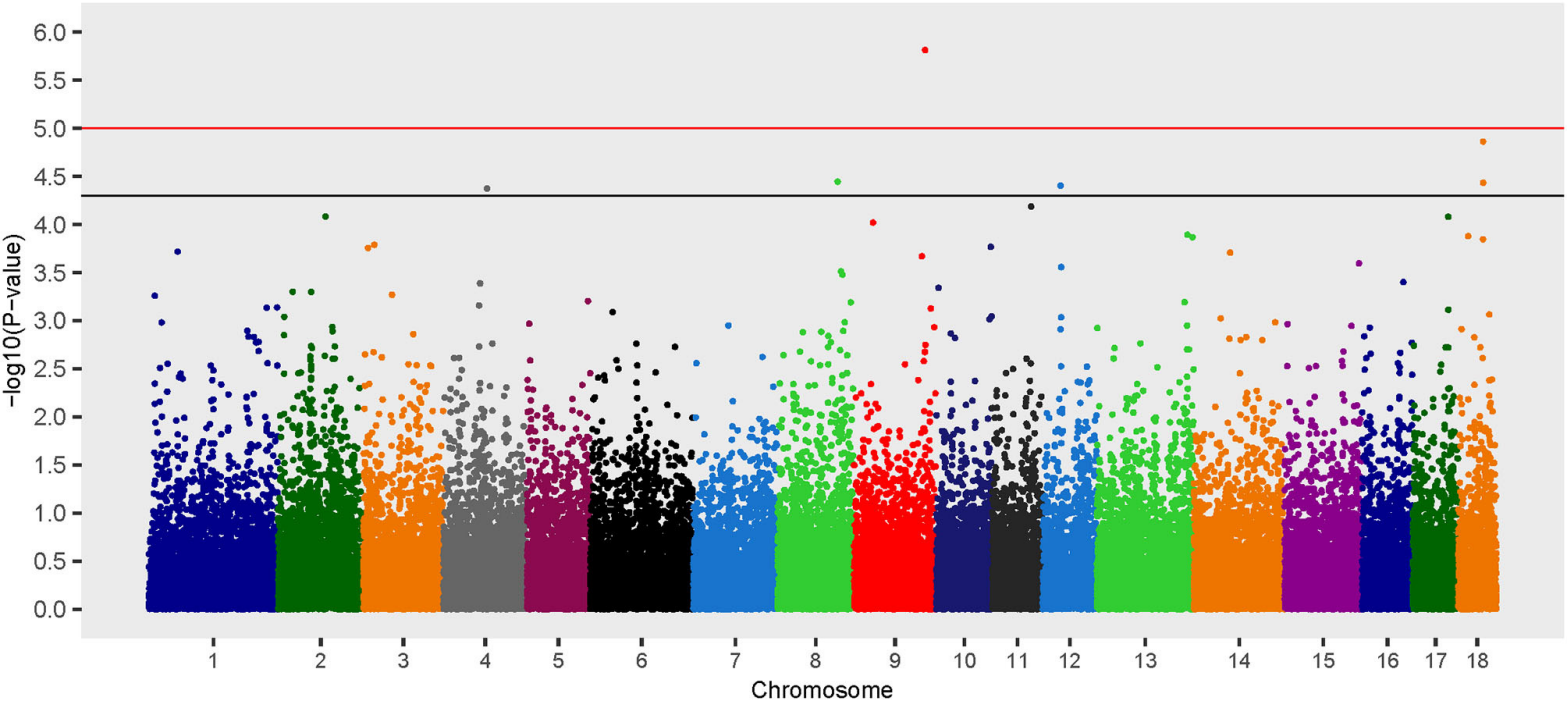
Manhattan plot of genome-wide association study for seroconversion following *Brucella suis* infection in feral swine using the EMMAX-GRM dominant model. The black line [–log_10_(*P*-value) = 4.3] denotes an association and the red line [–log_10_(*P*-value) = 5.0] denotes a moderate association with *B. suis* according to guidelines set forth by the Wellcome Trust Case Control Consortium (53).

Six loci on chromosomes 4, 8, 9, 12, and 18 were associated with *B. suis* seroconversion (Figure 2). Of the six loci, two were previously identified using the additive model: rs339122633 (*PTGS2*) and rs81469187 (*AOAH*). Three of the four SNP identified exclusively using the dominant model had *OR* < 1 which suggests that the major alleles at these loci (rs81404101 [major allele G], rs81329776 [major allele C], and rs338961194 [major allele G]) were associated with a decreased likelihood of *B. suis* seroconversion (Table 2). In contrast, the major allele (T nucleotide) for rs80941838 on chromosome 4 had an *OR* > 1 which suggests an increased likelihood of *B. suis* seroconversion. The two Mb genomic regions surrounding the significant SNP contained 55 annotated genes and 33 uncharacterized genes. Five putative candidate genes were identified after an extensive review of gene function and brucellosis infection: (1) Integrin Subunit Beta 3 (*ITGB3*), (2) Lysozyme Like 6 (*LYZL6*), (3) Mitogen-Activated Protein Kinase 14 (*MAP3K14*), (4) Tetraspanin 5 (*TSPAN5*), and (5) LYN Proto-oncogene, Src Family Tyrosine Kinase (*LYN*).

#### ITGB3

Integrins are transmembrane receptors important for cell adhesion and can be exploited by a number of pathogens for binding to host cells and internalization (82). ITGB3, encoded by the *ITGB3* gene, is a fibrinogen and vitronectin receptor that is expressed on platelets and monocytes with diverse roles in cell migration, adhesion, and signaling (83–85). ITGB3 (also known as αVβ3) can bind to adhesive proteins resulting in endothelial cell migration, angiogenesis, and TGF-B1 signaling (86).

Prostaglandin E2 suppresses the expression and activity of ITGB3 in human endometrial epithelial and stromal cells (87), suggesting a possible link with Prostaglandin-Endoperoxide Synthase 2, another gene identified in the current study. ITGB3 is also a marker and regulator of cellular senescence, a process that prevents propagation of damaged cells in tissue. In human primary fibroblasts under *in vitro* conditions, ITGB3 accelerates the onset of senescence by activating transforming growth factor beta [TGF-B; (88)]. Although senescent cells are metabolically active, the immune system senses these cells and eliminates them. The observed SNP could also have influenced N-glycosylation sites as these sites play a key role in regulating adhesive functions of integrins (89). Loss of certain individual N-glycan sites either reduce or enhance integrin activation, indicating that N-linked glycosylation can exert both positive and negative effects on integrin function.

Although the connection between *ITGB3* and reduced humoral responses was not evident, a possible link is with prostaglandin synthase, cellular senescence with immunologic elimination of targeted cells, or the role of this integrin in cell-to-cell adhesion and communication are all possible mechanisms.

#### LYZL6

Lysozymes are proteins that catalyze the hydrolysis of peptidoglycan on the bacterial cell wall. This results in bacteriolysis and the release of bacterial products, including cell wall peptidoglycan, which activates pattern recognition receptors in host cells (90, 91). Phagocytosis is an important innate immune function, which results in the fusion of bacteria within phagosomes with lysosomes and leads to acidification and degradation through proteases and oxidants. Through a poorly understood mechanism, *Brucella* spp. that are localized within phagosomes prevent fusion with lysosomes (92, 93). However, almost 90% of internalized bacteria in *Brucella*-containing phagosomes are killed, most likely through cellular processes such as acidification and oxidation that results from fusion with lysosomes (94). In antigen-presenting cells (especially dendritic cells), the bactericidal process could lead to expression of cleaved bacterial products on the phagocytic cell surface through exogenous antigen processing.

A probable hypothesis for the reduced likelihood of seroconversion associated with *LYZL6* would be that the compromised lysozyme results in reduced hydrolysis of the bacterial cell wall. This would result in peptidoglycan not being cleaved for antigen presentation and stimulation of cellular activation processes that result from interaction with pathogen pattern receptors.

#### MAP3K14

Mitogen-activated protein kinase 14 (*MAP3K14*) encodes NF-κB inducing kinase, a family of transcription factors that play important roles in the regulation of various cellular processes including cell growth, cell survival, cell development, and many aspects of immune function [e.g., immune responses and inflammation; (95–97)]. There are two major signaling pathways by which NF-κB activation can occur: canonical and non-canonical. Both are involved in immune stimulation and regulation; however, the non-canonical pathway functions include formation and architecture of secondary lymphoid organs, humoral immunity, dendritic cell maturation, and osteoclast and T cell differentiation. Additionally, the role of the non-canonical pathway in inflammatory diseases has been studied including rheumatoid arthritis, systemic lupus erythematosus, nephropathy, metabolic inflammation, and multiple sclerosis [reviewed in (98)].

While both pathways function in immunity and inflammation, the non-canonical pathway appears to be more selective than the canonical pathway, which could be partly attributed to the restricted type of receptors that can trigger its activation. However, certain pathogens have been shown to induce activation of the non-canonical NF-κB pathway including influenza virus (99), vesicular stomatitis virus (100), respiratory syncytial virus (101), and various herpesviruses (102–104). Some bacteria, such as *Helicobacter pylori* (105) and *Legionella pneumophila* (106), are also capable of activating the non-canonical pathway. Manipulation or modulation of host cell signaling serves as a virulence mechanism, which could enhance pathogen survival. Although some studies have shown the role of the canonical NF-κB pathway during *Brucella* infection (107, 108), there is no available information on the role of the non-canonical pathway during *Brucella* infection.

#### TSPAN5

Tetraspanins are conserved proteins that span the membrane of eukaryotic cells as membrane scaffolds, bringing together surface molecules such as integrins and cell-specific receptors into plasma membrane microdomains (109–112). Through their function as molecular scaffolds, tetraspanins contribute to development, reproduction, intracellular trafficking, and immunity (113, 114).

Our analysis identified a SNP in Tetraspanin 5, a gene that encodes TSPAN5 - a broadly distributed protein with reported physiologic functions in neurons, cartilage, osteoclasts, and the cardiovascular system (115–118). TSPAN5 is a member of a subgroup of tetraspanins (TspanC8) that interact closely with ADAM10 and regulate several functional aspects. Exit of ADAM10 from the endoplasmic reticulum, trafficking to either late endosomes or the plasma membrane, and specificity of ADAM10 relative to positive or negative regulation of Notch signaling is all modulated by TSPAN5 (111). ADAM10 is a transmembrane metalloproteinase that is responsible for cleaving off the ectodomain of various transmembrane proteins, which allows the intracellular domain to enter the cell nucleus and modulate gene expression, including those of cytokines (111, 119). Dendritic cells from mice in which ADAM10 has been knocked out have dramatic reductions in IgE production and do not develop significant TH2 immune responses (120). A role for ADAM metalloproteinases, sometimes through activation of Notch 1, has been demonstrated in both B cells (thymocyte and B cell development, function, antigen presentation), T cells (proliferation, activation, expression of CD44 adhesion molecules) and activation of NK cells suggesting an important role for this class of proteases in immune function [reviewed in (121)]. The role of *TSPAN5* in reduced humoral responses in feral swine after *Brucella* infection is not yet known, but it could be hypothesized that the effect may have been mediated through reduced activation of ADAM metalloproteinases.

#### LYN

Tyrosine-protein kinase Lyn, encoded by the *LYN* gene, is a member of the Src family of intracellular membrane-associated tyrosine kinases (SFK). Regulation of Lyn signaling is mediated by protein interactions through its Src homology (SH) domains, SH2/SH3, and its phosphorylation status. Although originally identified within the hematopoietic compartment, Lyn is expressed in many tissues and transmits signals from a variety of receptors, including the B cell receptor (122, 123). Downstream, Lyn can phosphorylate various targets including immunoreceptor tyrosine-based inhibitory/activation motifs (ITIM/ITAM) (124, 125), PI3 kinase (126), STAT5 (127), and MAP kinases, among others, resulting in both positive and negative regulatory signals. Lyn deficiency and Lyn overexpression both result in defects in the myeloid and lymphoid systems (122, 123, 128, 129).

In B cells, Lyn is the main SFK and has both positive and negative roles in B cell receptor signaling (124, 130). Within the BCR complex, Lyn is associated with IgM/IgD as well as CD19, and it is rapidly phosphorylated upon BCR cross-linking. Lyn can also positively and negatively influence toll-like receptor 4 (TLR4) signaling, a pattern recognition receptor, which recognizes bacterial LPS and plays a critical role in the initiation of innate immune responses (131). Lyn acts on TLR4 signaling, in part, by interfering with the activity of interferon regulatory factor-5 (IRF5), a molecule central to the downstream signaling of TLRs (132). In murine models, macrophages deletion of Lyn is associated with greater production of TNF-α, IL-6, and type I interferons after LPS stimulation (133).

Our data would suggest that the *LYN* gene may be involved in regulation of the humoral response to *Brucella* infection. Due to the role of Tyrosine-protein kinase Lyn in both BCR and TLR4 signaling, its effects could be mediated directly on B cells or through activation of innate immune danger signals. The crucial function of Lyn as both an activator and regulator of B cell responses, makes it an interesting target given the observed humoral phenotype in this study.

### Recessive Model

**Figure 3:**
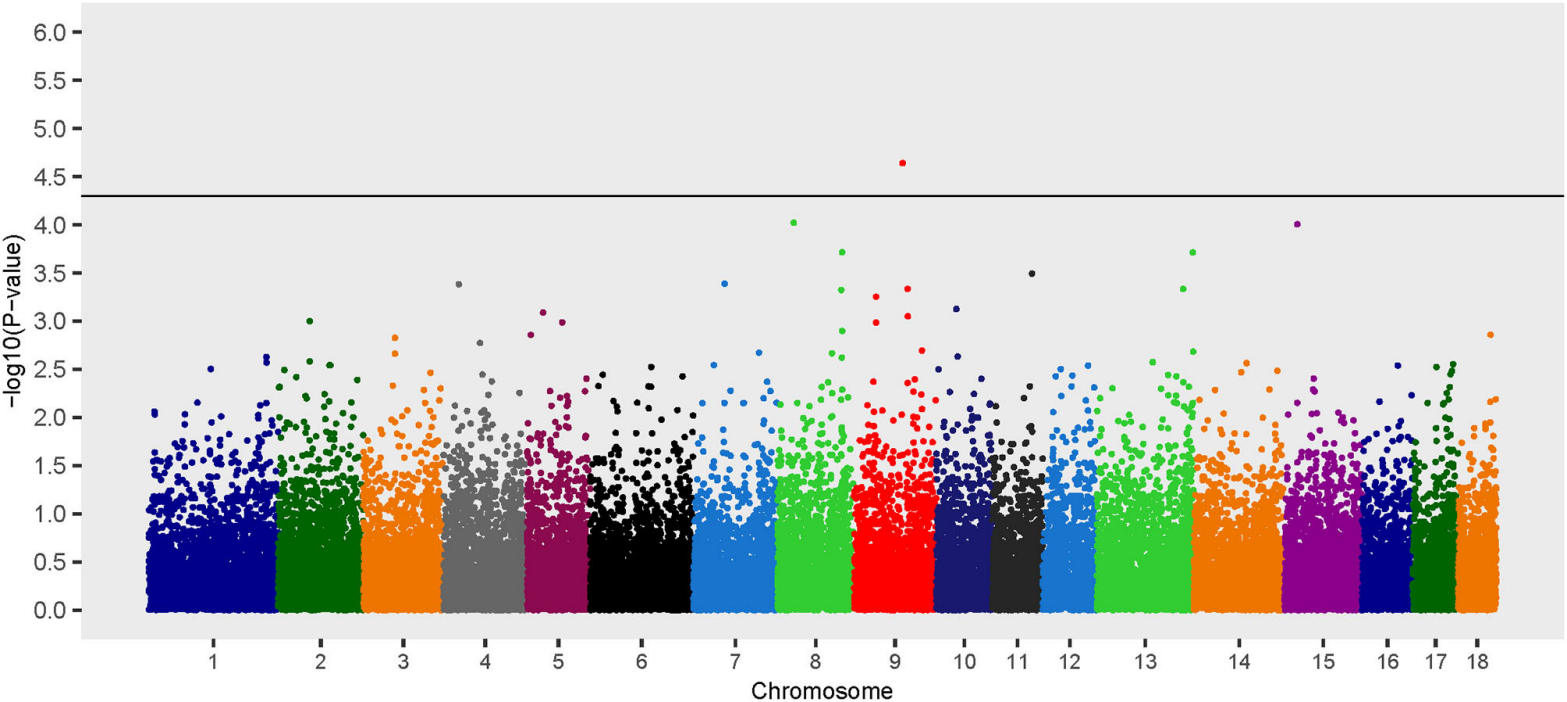
Manhattan plot of genome-wide association study for seroconversion following *Brucella suis* infection in feral swine using the EMMAX-GRM recessive model. The black line [–log_10_(*P*-value) = 4.3] denotes an association with *B. suis*.

The recessive model revealed one locus associated with *B. suis* seroconversion (Figure 3). The odds ratio for the major allele (nucleotide T) at this locus (rs81413617) was 0.30, which indicates that this allele is associated with a decreased likelihood of *B. suis* seroconversion (Table 2). Seven uncharacterized genes as well as 10 annotated genes were located within the two Mb genomic region encompassing this SNP. The SNP, rs81413617, was located on chromosome 9 within an uncharacterized gene *LOC110262233*; however, one of the genes located 0.35 Mb upstream of rs81413617 was linked to immune response: Collagen Type XXVIII Alpha 1 Chain (*COL28A1*).

#### COL28A1

The collagen superfamily covers a variety of subclasses and collagens that can be found in all tissues throughout the vertebral body and are important for tissue integrity (134). COL28A1 collagen is believed to have greatest expression in the nervous system and encodes a von Willebrand factor A domain that facilitates protein-protein interactions. Genome-wide association analyses have previously identified an association between this type of collagen with Interleukin 1 beta (IL-1β) secretion in African Americans following smallpox vaccination (135) and resistance to clinical mastitis in cattle (136). The gene was also found to be down regulated in a rat model of lipopolysaccharide-induced epididymitis (137) and upregulated in a bleomycin lung injury murine model (138).

Current knowledge does not readily explain how *COL28A1* would influence humoral responses to *B. suis* in pigs; however, the possible tie to IL-1β secretion is of interest. IL-1β plays a crucial role in modulating host immune responses to inhaled pathogens through expression of chemokines and adhesion molecules, enhancing phagocytic activities of neutrophils and monocytic cells, and increasing production of reactive oxygen species (139, 140). In addition, mice lacking IL-1β receptors have increased pulmonary colonization after intratracheal infection with *Brucella abortus* (141).

## Discussion

In evaluating the physiological response of feral swine to *B. suis* infection with case-control GWAS, we identified eight variants and 145 positional candidate genes suggesting seroconversion following *B. suis* infection of feral swine may be under polygenic control. A review of the current understanding of gene function and infection kinetics reduced the number of plausible candidate genes involved in the immunologic response of feral swine to *B. suis* from 145 to nine. This aligns with previous studies that evaluated the genetic architecture of immune response in domestic swine (39, 142, 143). The nine genes that were thoroughly explored were all related to immune function—most notably phagocytosis and induction of an inflammatory response. *Brucella* spp. are intracellular bacteria that utilize various methods to evade and modulate the host immune response. The identified loci and putative candidate genes may play an important role in host immune responses to *B. suis* infection, characterized by a detectable bacterial presence yet a differential antibody response.

The candidate loci mapped to non-coding regions of the swine genome, suggesting involvement in regulation of gene expression. Non-coding variants can modulate transcription factor binding within promoter and enhancer regions, methylation, targeted recruitment of transcriptional activators and repressors, gene splicing, microRNA (miRNA) binding to 3′UTR, and expression of long non-coding RNAs [lncRNA; (144–147)]. Studies suggest that changes in gene expression can dysregulate immune pathways that are fundamental in disease outcomes (146, 148). For instance, SNP found within cytokine promoters have been linked to susceptibility to *Mycobacterium tuberculosis*—a pathogenic intracellular bacteria that causes tuberculosis (149).

Several genes associated with host resistance or susceptibility to *Brucella* spp. have been identified in cattle, goats, and humans. For example, previous studies have reported associations between SNP in natural resistance-associated macrophage protein 1 (*NRAMP1*) and *B. abortus* resistance or susceptibility in cattle (150–152). Another gene previously linked to *B. abortus* in cattle is Toll-like receptor 4 [*TLR4*; (152)]. In goats, Rossi et al. (153, 154) associated an intronic polymorphism (rs657542977) in protein tyrosine phosphatase receptor type T (*PTPRT*) and an insertion/deletion polymorphism (InDels; rs660531540) in interferon regulatory factor 3 (*IRF3*) with host resistance. A study of human patients infected with *Brucella* revealed a SNP within interferon gamma (*IFNG*) associated with increased susceptibility to *B. melitensis* (155). However, brucellosis is caused by multiple species of *Brucella* that exhibit preferential host specificity [e.g., *B. abortus* [cattle, bison, and buffalo], *B. melitensis* [sheep and goats], *B. suis* [pigs], *B. ovis* [sheep], *B. canis* [dogs], *B. neotomae* [mice], *B. pinnipedia* [seals, sea lions, walruses], and *B. cetacea* [whales, porpoises and dolphins]; (156, 157)]. Therefore, brucellosis disease dynamics may differ in swine relative to other species due differences among hosts and *Brucella* spp.

Although genes previously associated with *Brucella* spp. resistance/susceptibility are conserved across species, we did not find associations of these genes in our survey of naturally infected feral swine. This discordance may be due to the fact that our analysis focused on the seroconversion of culture-positive animals as opposed to resistant (non-infected) or susceptible (infected) phenotypes such as IFN-γ responses. Also, the genotyping array used in this study did not contain SNP within three of the five genes previously associated with brucellosis infections. Future research could use targeted resequencing to evaluate the influence of genes previously associated with immune response to *Brucella* spp. that were not effectively evaluated with this SNP array.

With this analysis we identified statistical associations between loci and seroconversion in infected feral swine; however, GWAS are ineffective for determining the causal variant and the mechanism in which the variant affects biological pathways contributing to seroconversion (158, 159). Further, our limited sample represented naturally infected animals opportunistically identified from a broader disease surveillance study (9). Therefore, follow-up experimental infection studies are needed to fully elucidate the genetic drivers of differential host responses to swine brucellosis and provide additional understanding of epidemiological processes.

Although brucellosis was eradicated from U.S. commercial swine, in some regions of the U.S. there has been a shift from biosecure commercial operations to pasture systems, thus, presenting an increased opportunity for disease spillover through contact with feral swine. Re-emergence of *B. suis* in domestic pigs would have enduring effects on production systems and the pork export market. Currently, the prevalence of brucellosis among feral swine, and the concomitant risk of disease transmission to domestic pigs, is interpreted through serological assays as a component of a nationwide disease surveillance program. The underestimation of infection using routine serological diagnostics is problematic and creates substantial challenges in estimating true disease prevalence, which is critical for effective management (160). Understanding genetic determinants of seroconversion following infection with *B. suis* across the invaded range would enable better interpretation of serologic diagnostic results, optimal allocation of surveillance efforts, and focused elimination efforts on feral swine populations that pose the greatest risk of spillover to domestic pigs (161).

## Data Availability Statement

Data presented in this study are available through the European Variation Archive under accession: PRJEB40856.

## Ethics Statement

Ethical review and approval was not required for the animal study because previously published data was used (9) and genotyping was conducted ancillary to sample collection.

## Author Contributions

CP and TS contributed to the development of the idea, collection of the data, data analysis and interpretation, and preparation of the document. VB and SS contributed to the development of the idea, data analysis and interpretation, and preparation of the document. SO and PB contributed to data analysis and interpretation and preparation of the document. KP contributed to collection of the data, data analysis and interpretation, and preparation of the document. RM contributed to data analysis and preparation of the document. All authors contributed to the article and approved the submitted version.

## Conflict of Interest

The authors declare that the research was conducted in the absence of any commercial or financial relationships that could be construed as a potential conflict of interest.
