# Supplementary material for: Loci Associated With Antibody Response in Feral Swine (Sus scrofa) Infected With Brucella suis
Source: Front Vet Sci. 2020 Nov 25;7:554674. doi: 10.3389/fvets.2020.554674 (PMC7724110; doi:10.3389/fvets.2020.554674)
Supplement: Supplementary file 1 [file Data_Sheet_1.docx]

Supplementary Material

# Supplementary Methods

Misclassification of the serological status of an animal could result from two potential sources of error: 1) error due to diagnostic test performance (sensitivity and specificity) or 2) the physiological delay in an animal’s immune response once infected with *B. suis* resulting in a false negative result. We evaluated both potential sources of sampling error.

## Misclassification due to diagnostic test performance

The probability of an individual being misclassified as serologically negative when they are positive due to diagnostic test performance can be calculated as the combined probability of having more than six negative serological test results in any combination. To determine this probability, we conservatively used the lowest previously published sensitivity value for each serological diagnostic test (Table S1; Fosgate et al., 2002; Nielsen, 2002).

## Misclassification due to physiological delay in immune response

### Estimation of serum antibody response

False negative misclassification resulting from the delay in serologic response was assessed using data describing the serological response of an infected animal from time to infection until 120 days post infection. Data describing a pigs serum antibody response for competitive enzyme‐linked immunosorbent assay [cELISA], complement fixation [CFT], fluorescence polarization assay [FPA], Serum (tube) agglutination test [SAT], and plate agglutination [PA] were acquired from Jungersen et al. (2006). Data describing test response for buffered acidified plate antigen test [BAPA] and card test used experimental infections conducted at National Veterinary Services Laboratory (Brown et al., 2020). Data describing the serum antibody response through time for the rivanol test could not be identified. However, the rivanol test detects principally IgG1 and, to a lesser extent, IgG2. Similarly, complement fixation detects principally IgG1, thus the rivanol test was assumed to respond in a similar way through time and was used as a proxy for the rivanol test (Mikolon et al. 1998).

Bayesian generalized additive models (GAM) were fit to the referenced serum antibody response data (response variable) resulting in a posterior distribution describing the daily (predictor variable) change in serum antibody response for each diagnostic test. In the case of plate agglutination, buffered acidified plate antigen test [BAPA] and the card test the response data was a binary (positive or negative) resulting in a posterior distribution that predicted the probability of testing positive on any given day. The GAM model was formulated as:

$y_{i}=f\left( x_{i} \right)$.

where $y_{i}$ is the serum antibody response or the binary test result on day $i$ and $f\left( x_{i} \right)$ is the smoothing function that relates the change in serum antibody response to the day post infection. The error structure used for each diagnostic test is described in Table S1 and represents the scale of the response data. Posterior distributions were predicted using 10,000 samples with a burn in of 2,500 samples. The optimal smoothing function, $f\left( x_{i} \right)$, was selected based on the lowest Akaike information criterion (AIC). The resulting fitted models are presented in Figure S1 and S2. Models were fit in the R commuting environment (Team 2013) using the Bayesian Regression Models and the Stan (brms) R package (Bürkner 2017).

### Simulation of diagnostic test results

Posterior distributions (Figure S1 and S2) for the response of each serological test were used to simulate 1) the probability of a positive classification (at least two positive test results) for each day post infection and 2) the probability of a true positive and false negative classification for animals included in this study.

### Daily probability of positive test result

To determine the daily probability of a positive test result, the posterior distribution for each diagnostic test was randomly sampled for an individual animal for each day. The test results for the simulation were determined based on the cut-points for the diagnostic test and the animal was classified as either positive or negative. This was done for 10,000 iterations resulting in a derived posterior distribution of infected and uninfected animals for each day post infection from day 0 to day 120. The posterior probability of a positive test result can then be calculated as,

$$Pr\left( positive|testing \right)=\frac{1}{n_{j}}\sum_{j=0}^{J} \theta_{j}$$

$$\theta_{j}={\{}_{0 otherwise}^{1 if postive}$$

Where $n_{j}$ is the number of simulated draws from the posterior distribution on day $j$ and $\theta_{j}$ is the resulting classification (positive or negative) resulting from the eight diagnostic tests. The posterior probability of a true positive classification by day is presented in Figure S3.

### Sample probability of true positive and false negative classification

The day of infection is unknown for animals included in this study, thus a calculation of the sample probability of true positive and false negative classification is useful in understanding the limitations of the sample population. It was assumed that the 47 positive animals in the study were exposed to *B. suis* sometime in the last 4 months (120 days). Assuming these 47 animals were randomly assigned a day $j$ post-infection, the posterior distribution for each diagnostic test was randomly sampled for animal $i$ on day $j$ and if two or more tests were positive then the animal was classified as positive. The probability of true positive classification for the sample was then calculated as:

$$Pr\left( true positive classification|testing \right)=\frac{1}{47}\sum\theta_{i}$$

$$\theta_{i}={\{}_{0 otherwise}^{1 if postive}$$

where $\theta_{i}$ is the simulated classification of animal $i$. This was done 10,000 times and the mean across all 10,000 iterations was used to estimate the probability of true positive classification.

# Supplementary Tables

Table S1. Performance, false negative probabilities, and false positive probabilities for the eight serological tests used in this study (Fosgate et al., 2002; Nielsen, 2002).

| **Serological**  **Test** | **Sensitivity**  **Range** | **Sensitivity** | **Specificity**  **Range** | **Specificity** | **Pr(False Neg)** | **Pr(False Pos)** | **Pr(False Neg)**  **(Low Sensitivity)** |
| --- | --- | --- | --- | --- | --- | --- | --- |
| BAPA | 76.2–95.6 | 85.9 | 96.4–99.3 | 97.85 | 0.1410 | 0.0215 | 0.238 |
| cELISA | 97.5–100 | 98.75 | 99.7–99.8 | 99.75 | 0.0125 | 0.0025 | 0.025 |
| CFT | 23.0–97.1 | 60.05 | 30.6–100 | 65.30 | 0.3995 | 0.3470 | 0.77 |
| FPA | 99.0–99.3 | 99.15 | 96.9–100 | 98.45 | 0.0085 | 0.0155 | 0.01 |
| Rivanol | 50.5–100 | 75.25 | 21.9–100 | 60.95 | 0.2475 | 0.3905 | 0.495 |
| PAT | 50.9–80.4 | 65.65 | 97.5–99.6 | 98.55 | 0.3435 | 0.0145 | 0.491 |
| STT | 29.1–100 | 64.55 | 99.2–100 | 99.60 | 0.3545 | 0.0040 | 0.709 |
| Card | 74.3–99.0 | 86.65 | 7.4–100 | 53.7 | 0.1335 | 0.4630 | 0.257 |

Table S2. Error structure used in generalized additive models.

| **Diagnostic Test** | **Model** |
| --- | --- |
| Enzyme‐linked immunosorbent assay [cELISA] | Gaussian |
| Complement fixation [CFT] | Log Normal |
| Fluorescence polarization assay [FPA] | Gaussian |
| Plate agglutination [PA] | Bernoulli |
| Serum (tube) agglutination test [SAT] | Log Normal |
| Buffered acidified plate antigen test [BAPA] | Bernoulli |
| Card test | Bernoulli |

# Supplementary Figures

Figure S1. Predicted relationship between serum antibody response and day post infection. Black line indicates mean predicted response and gray band is the 95% credible interval. Horizontal dashed line indicates the establish cut-point for determination of a positive serologic determination.

.
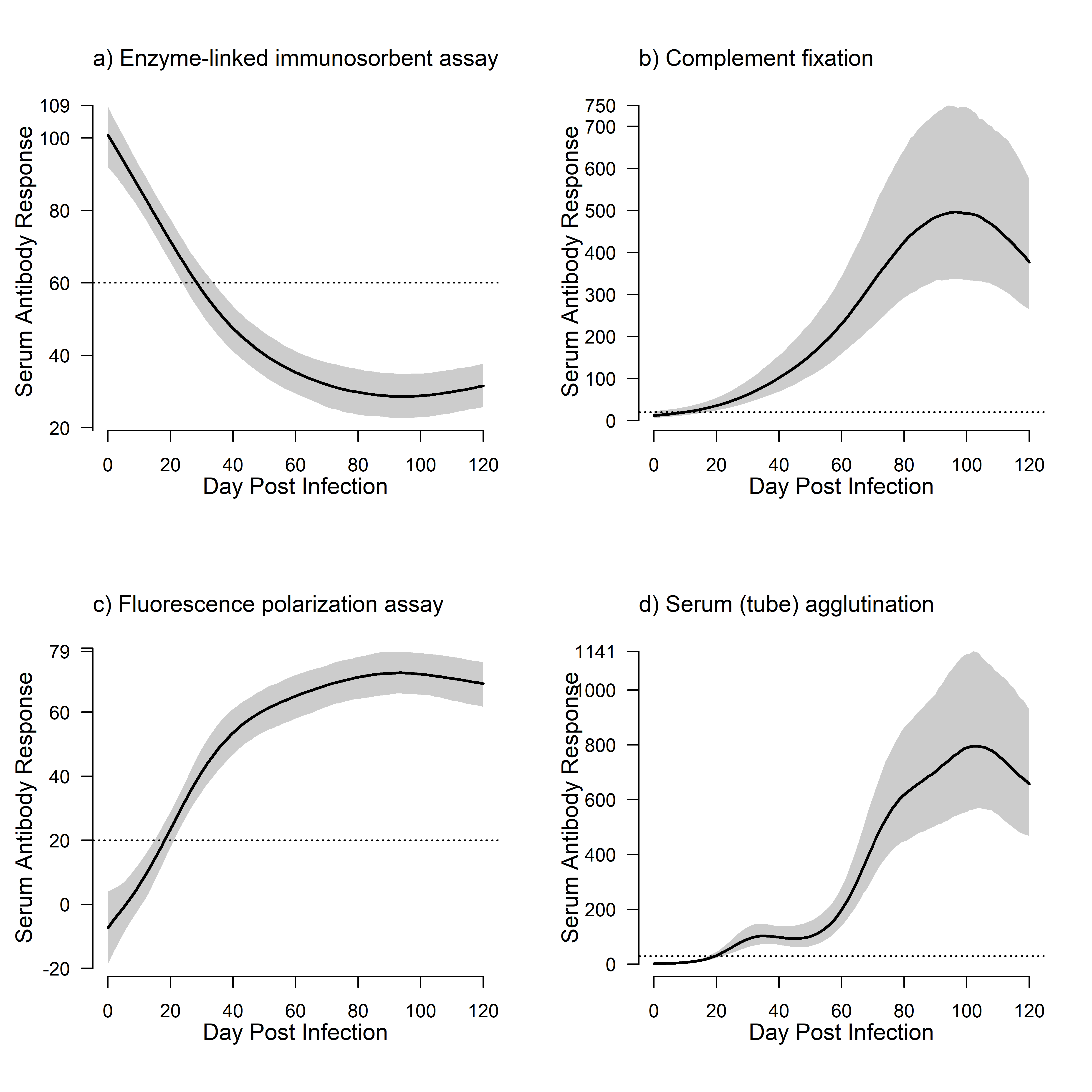


Figure S2. Predicted relationship between the probability of a positive test result and day post infection. Black line indicates mean predicted response and gray band is the 95% credible interval.

.
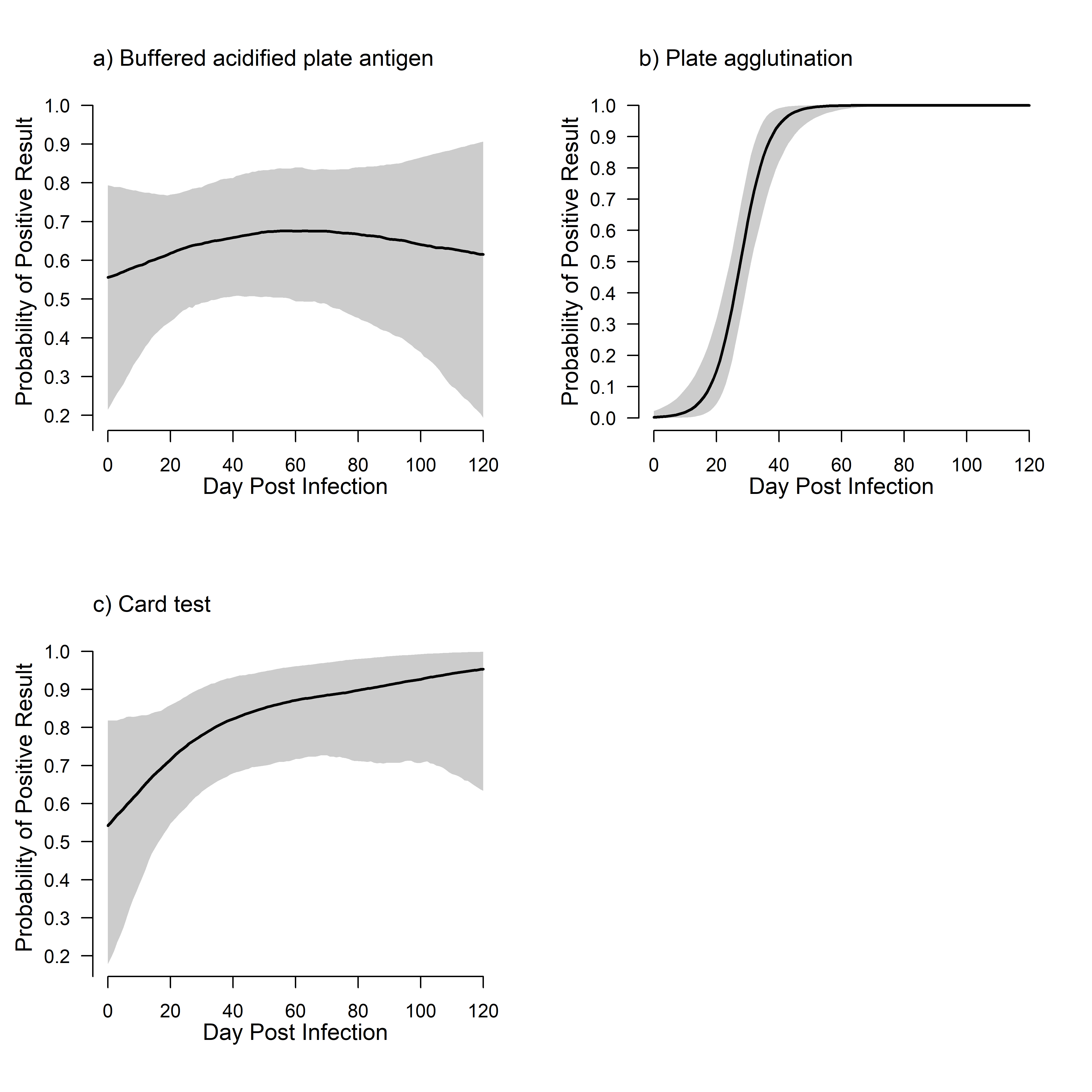


Figure S3. Posterior predicted probability of positive serologic test classification assuming two or more positive tests.


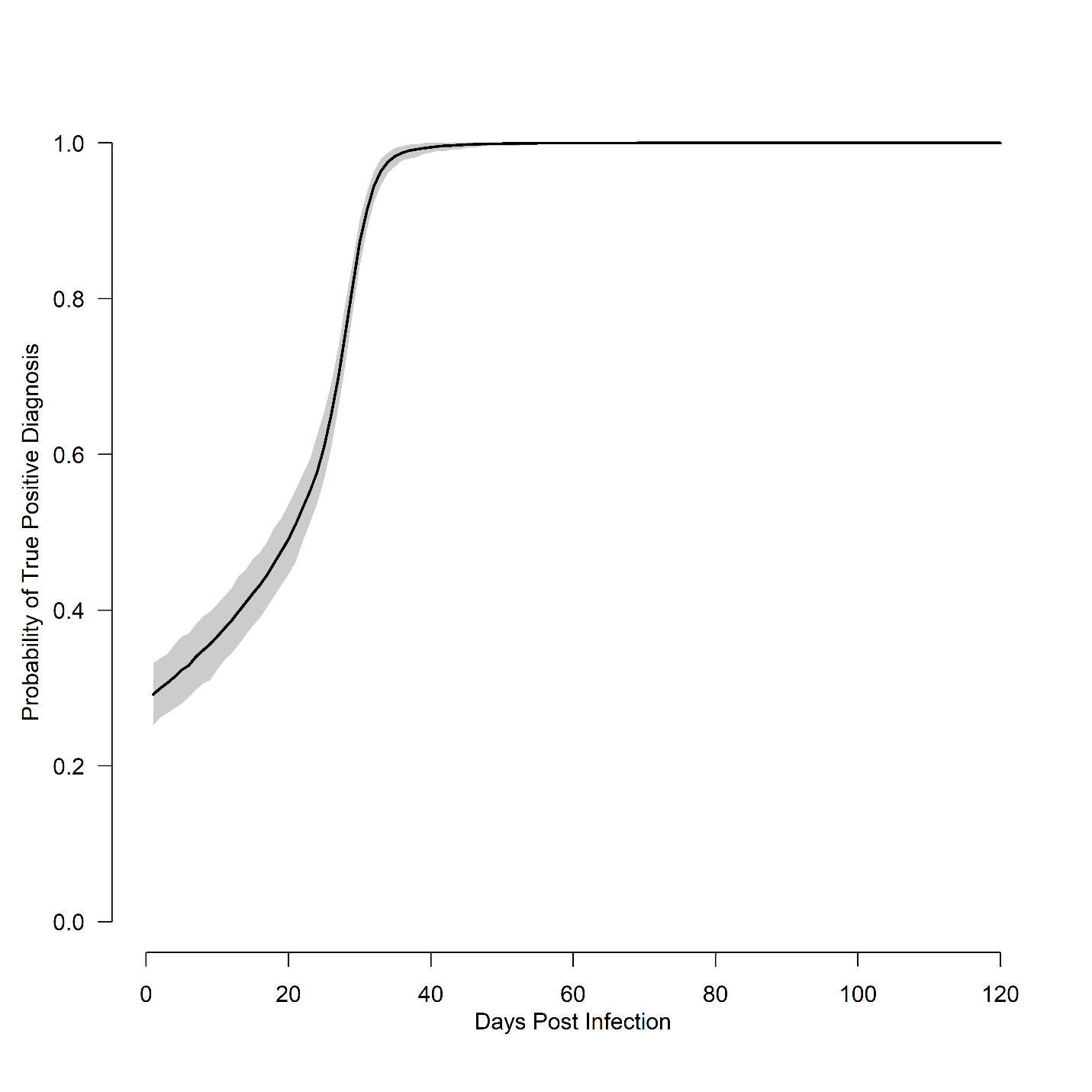


# References

Brown, V.R., Bowen, R.A., Ledesma, N., Hartwig, A., Gordy, P., Pierce, C.F., Anderson, A., Porter, S., Alexander, K., Goucker, Z., Ragan, I., Gidlewski, T., Bosco-Lauth, A. (2020). Pathogenesis and Immune Responses of Heritage Breed Pigs to Experimental Inoculation with Brucella suis. [Manuscript submitted for publication]. Department of Biomedical Sciences, Colorado State University.

Bürkner, P. 2017. Bayesian Regression Models using Stan. R package version 1.

Jungersen, G., Sørensen, V., Giese, S., Stack, J., Riber, U. (2006). Differentiation between serological responses to Brucella suis and Yersinia enterocolitica serotype O[ratio]9 after natural or experimental infection in pigs. Epidemiol. Infect. 134(2), 347-357. doi:10.1017/S095026880500511XMikolon, A. B., I. A. Gardner, S. K. Hietala, J. H. De Anda, E. C. Pestaña, S. G. Hennager, and A. J. Edmondson. 1998. Evaluation of North American antibody detection tests for diagnosis of brucellosis in goats. Journal of clinical microbiology 36:1716-1722.

Team, R. C. 2013. R: A language and environment for statistical computing. Vienna, Austria.
